# Precision Phenomapping of Acute Coronary Syndromes to Improve Patient Outcomes

**DOI:** 10.3390/jcm10081755

**Published:** 2021-04-18

**Authors:** Felicita Andreotti, Adelaide Iervolino, Eliano Pio Navarese, Aldo Pietro Maggioni, Filippo Crea, Giovanni Scambia

**Affiliations:** 1 Catholic University of the Sacred Heart, 00168 Rome, Italy; adelaide.iervolino@libero.it (A.I.); filippo.crea@unicatt.it (F.C.); giovanni.scambia@policlinicogemelli.it (G.S.); 2 Departments of Cardiovascular and Personalised Medicine, Fondazione Policlinico Universitario A. Gemelli IRCCS, 00168 Rome, Italy; 3 Interventional Cardiology and Cardiovascular Medicine Research, Department of Cardiology and Internal Medicine, Nicolaus Copernicus University, 85094 Bydgoszcz, Poland; elianonavarese@gmail.com; 4 Faculty of Medicine, University of Alberta, Edmonton, AB T6G 2R3, Canada; 5 SIRIO MEDICINE Research Network, 85094 Bydgoszcz, Poland; 6 ANMCO Research Center, Fondazione per il Tuo cuore, 50121 Florence, Italy; maggioni@anmco.it; 7 GVM Care & Research, Maria Cecilia Hospital, 48033 Cotignola, Italy

**Keywords:** acute coronary syndromes, precision, phenomapping, omic technologies, treatments, outcomes

## Abstract

Acute coronary syndromes (ACS) are a global leading cause of death. These syndromes show heterogeneity in presentation, mechanisms, outcomes and responses to treatment. Precision medicine aims to identify and synthesize unique features in individuals, translating the acquired data into improved personalised interventions. Current precision treatments of ACS include immediate coronary revascularisation driven by ECG ST-segment elevation, early coronary angiography based on elevated blood cardiac troponins in patients without ST-segment elevation, and duration of intensified antithrombotic therapy according to bleeding risk scores. Phenotypically stratified analyses of multi-omic datasets are urgently needed to further refine and couple the diagnosis and treatment of these potentially life-threatening conditions. We provide definitions, examples and possible ways to advance precision treatments of ACS.

## 1. Introduction

Ischaemic heart disease is a leading cause of death globally, and acute coronary syndromes (ACS) are among its most severe and life-threatening manifestations [1,2]. The implementation of international guidelines [3,4,5] has led to improved patient outcomes [6,7]. Guideline recommendations, however, are largely based on trials conducted in “typical” populations. This approach may have exhausted, at least in part, its ability to further improve the natural history of ACS patients considered as a whole [8]. Certain subgroups still have an enhanced risk of early death or other severe complications, and the challenge of future research is to identify such patients [8]. Traditional predictive scores and, more recently, machine learning algorithms are being proposed to stratify patients at different levels of risk [9]. Drastic potentiation of deep precision mapping of ACS and of healthy phenotypes is also needed.

## 2. Precision and Personalised Medicine

Precision medicine subdivides common diseases into subgroups according to optimal treatment strategies, identifying patients who will benefit most in terms of efficacy and prognosis [8,10]. Ideally, for each patient, precision medicine rapidly and simultaneously synthesises data collected from traditional types of care (e.g., history, physical, imaging, laboratory) with those provided by omic technologies (e.g., metabolomic, proteomic, next-generation sequencing analyses enabling genome, transcriptome, DNA-protein interaction profiling) [11]. Precision and personalised medicine are often used interchangeably and are closely related [12]; precision medicine, however, is involved in *identifying and synthesizing* the unique features of an individual’s health condition, whereas personalised medicine is involved in *implementing* the acquired data *and administering* person-specific treatment plans [11]. In other words, precision is directed more to disease pathogenesis and personalised to patient management.

Lumping clinical presentations with common signs and symptoms into prespecified disease pathways all treated in the same way according to evidence-based medical guidelines (the so-called reductionist approach) [11,12] has led to a significant contraction of the overall burden of cardiovascular disease. The main advantage of this approach is the broad allocation of treatments proven to be effective and acceptably safe when tested in large and fairly similar populations [6,7]. Disadvantages are that patient specificities, including variable presentations, unpredictable outcomes, treatment unresponsiveness or side-effects, are not fully taken into account. While reductionists (‘lumpers’) tend to group entities together into oversimplified schemes, a ‘precisionist’ (‘splitter’) tends to collect data, emphasizing differences and unravelling complexities, in order to address the diversity of disease manifestations [11,12]. Although precision medicine splits, it also aims to recompose the entire clinical picture to reach more refined nosologies and patient-tailored treatments.

## 3. Why Intensify Precision Medicine for Acute Coronary Syndromes

ACS are a cluster of multifactorial diseases. This cluster is currently subdivided into ST-segment elevation myocardial infarction (STEMI), non-STEMI and unstable angina [3,4,5]. The pathoanatomy underlying many, but not all, of these syndromes (presenting typically with sudden prolonged chest pain along with electrocardiographic (ECG) and/or blood biomarker abnormalities) is a focal thrombotic occlusion or subocclusion of one or more epicardial arteries overlying a fissured or eroded atherosclerotic plaque [3,4,5,8,13,14]. According to current knowledge, atherothrombosis develops in response to prolonged exposure to multiple cardiovascular risk factors (dyslipidaemia, hypertension, diabetes, other prothrombotic and proinflammatory states), variably interacting with polygenic predisposition and unhealthy lifestyles [3,4,5,8,13,14]. Of note, relatively few monogenic disorders to date—most notably, familial hypercholesterolaemia, largely caused by mutations of the low-density lipoprotein receptor gene (*LDLR*)—have been identified as contributing significantly to the onset of ACS [15]. Depending on patient characteristics, severity of presentation and type/quality of acute management, the clinical outcomes can vary dramatically, ranging from prehospital sudden death, cardiogenic shock, electrical instability, recurrent infarction, heart failure, angina or adverse drug effects, at one end, to totally asymptomatic and uneventful courses, at the other [3,4,5]. Genetic and deep phenotypic precision mapping of these syndromes has the potential to unravel the heterogeneous presentations, anatomical substrates, treatment responses and natural histories of ACS, ultimately leading to focused therapies and improved outcomes.

## 4. Deep Precision Mapping of Acute Coronary Syndromes

**Figure 1:**
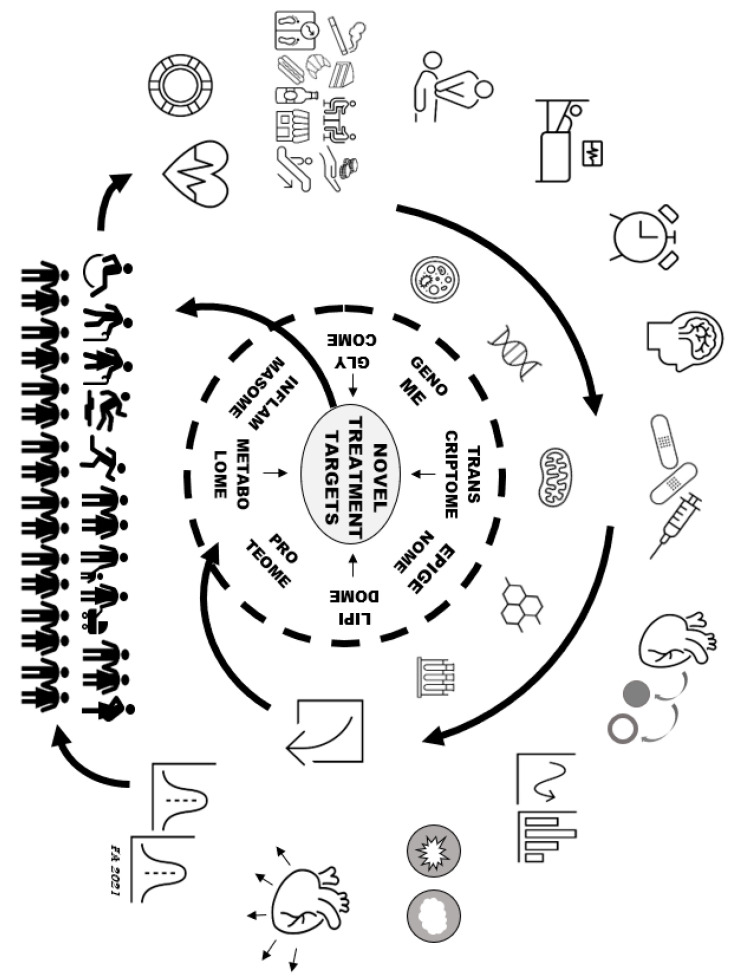
Deep precision mapping of ACS phenogroups. Outer circle: patients may be immediately stratified on the basis of non-omic features, such as age, sex, transient or persistent chest pain, ECG changes, suspected vasospasm, risk factor exposure, cardiac arrest, time of day of clinical onset, preinfarction angina, response to therapies, underlying plaque fissure or erosion, prothrombotic/inflammatory conditions, or adverse myocardial remodeling. Inner circle: biomolecular omic interrogation of the above different phenogroups is bound to expand knowledge of underlying mechanisms. Atypical presentations (tails of frequency curves) may reveal yet unrecognised forms of disease. Modified from [16]. ACS: acute coronary syndromes.

Splitting and recomposing ACS through precisional research can provide insights into the different mechanisms underlying apparently similar clinical presentations. These mechanisms may include epicardial artery thrombosis, dissection, plaque erosion, plaque rupture, vasospasm, microvascular disease or other pathways [8,13,14]; the inner biological networks and molecular crosstalk driving these different phenogroups are likely to be distinct. Figure 1 illustrates how the interaction between phenogrouping, on the one hand, and deep precision mapping, on the other, is bound to offer insight into the heterogeneity of ACS patients. Each subphenotype (defined largely on the basis of non-omic factors) is susceptible to interrogation using the non-omic and biomolecular omic precisional mapping tools listed in Table 1.

## 5. Current Precision Treatments of Acute Coronary Syndromes

The use of omic disciplines—globally referred to as panomic or multi-omic technologies—to support decisional processes may represent a daunting perspective for clinicians dealing with ischaemic heart diseases. Only recently have paradigms begun to shift**,** with big data collection starting to offer omic information to practicing cardiologists [17,18,19]. On the other hand, oncology, haematology, immunology and infectious diseases have been integrating these tools for some decades into what are now well-established diagnostic and treatment protocols [12].

Notably, omic data use is not the only way to apply precision medicine to ACS. Indeed, contemporary cardiologists already stratify ACS, selecting treatments accordingly. Examples are the ECG detection of persistent ST-segment elevation directing chest pain syndromes towards immediate coronary revascularisation [3], increased serum levels of cardiac troponins directing non-STEMI ACS towards an invasive, as opposed to a conservative, management strategy [4,20], or the use of predictive risk scores based on phenotypic-cluster analyses, such as the GRACE score for early mortality (age, cardiac arrest at admission, blood pressure, heart rate, ECG changes, myonecrosis markers, creatinine, rales, where values >140 direct toward more intensive monitoring) or the PRECISE-DAPT score for high bleeding risk (age, creatinine clearance, haemoglobin, leukocyte count, prior bleeding, where values ≥25 direct toward shortened dual antiplatelet therapy) [4,20]. Examples of established and investigational precision treatments of ACS patients are shown in Table 2.

## 6. Precision Research Applied to Acute Coronary Syndromes

To further illustrate the potential of precision medicine applied to ACS, several examples are provided.

A recent trial enrolled ACS patients with previous myocardial infarction according to baseline serum levels of the inflammatory marker C-reactive protein ≥2 mg/L, which had been previously shown to predict adverse outcomes [28]. Patients were randomised to the interleukin-1 beta monoclonal antibody, canakinumab, or placebo; canakinumab administered subcutaneously every 3 months over 3.7 years reduced cardiovascular death, myocardial infarction or stroke compared to placebo. Although costs and side effects prevented approval of this agent for this particular indication, the trial demonstrated a causal role of the interleukin-1 beta pathway for the development of cardiovascular complications in patients with previous myocardial infarction and raised circulating C-reactive protein [29].

Ongoing studies are focusing on prognostic biomarkers correlated with individual phenotypes. ‘The microbiome as a target for precision medicine in atherosclerosis (MIGATER)’ investigation is prospectively recruiting age-matched acute and chronic coronary syndrome patients to assess the role of dysbiosis in the progression of disease [30]. Microbiota, coupled with genetic, immunological and cytokine analyses, will be compared to clinical events and changes in fibrous cap thickness assessed by intravascular imaging. Studies supporting this approach include the independent association of elevated plasma trimethylamine N-oxide levels, a gut microbe-dependent metabolite, with the development of ACS [31]. A further study is the ‘biomarker-based prognostic assessment’ multicenter prospective registry, involving up to 10,000 patients, aiming to derive prediction models of ischaemic and bleeding events through strict follow-up of patients hospitalised for acute or chronic coronary syndromes. Individuals will be characterised by demographic, risk factor, blood sample, angiographic and interventional data [32].

In another trial (DalGenE), patients 4–12 weeks after experiencing an ACS who carry the rs1967309 adenylate cyclase type 9 genotype are being randomised to either dalcetrapib (a cholesteryl ester transfer protein inhibitor leading to high-density lipoprotein-cholesterol elevation) or placebo [27]. This gene variant was selected because it was previously found to be associated with reductions in cardiovascular risk or in carotid intima-media thickness after dalcetrapib compared to placebo treatment [33].

Finally, intracoronary optical coherence tomography conducted in STEMI patients and analysed in relation to the time of onset of infarction [34] has shown different underlying pathologies (plaque rupture, plaque erosion, calcified plaque) in 60%, 30%, and 10% of patients. The incidence of plaque rupture was found to peak in the morning, presumably related to catecholamine surge [34]; at this time of day, occlusive atherothrombosis is favoured by lower endogenous blood fibrinolytic activity [35].

## 7. Precision Mapping of Healthy Phenotypes

Mapping healthy phenotypes or groups protected from cardiovascular diseases is as important as mapping patho-phenotypes and has proven crucial for identifying cardiovascular disease targets. A recent example of such advances is the development of a new class of therapeutic agents based on the discovery of the gene encoding proprotein convertase subtilisin/kexin type 9 (PCSK9) as a regulator of LDL cholesterol and the discovery that loss-of-function variants in this gene protect against ischaemic heart disease [36].

In the large Atherosclerosis Risk in Communities general population [37], the incidence of nonfatal myocardial infarction, fatal coronary artery disease or coronary revascularisation over a 15-year interval was analysed according to the presence or absence of sequence variants in the PCSK9 gene (*PCSK9*) associated with reduced plasma levels of LDL cholesterol. Of the 3363 black subjects examined, 2.6% had nonsense mutations in *PCSK9*; these mutations were associated with a 28% reduction in mean LDL cholesterol and a significant 88% reduction in the risk of ischaemic heart disease. Of the 9524 white subjects examined, 3.2% had a sequence variation in *PCSK9* that was associated with a 15% reduction in LDL cholesterol and a significant 47% reduction in the risk of ischaemic heart disease. Thus, a moderate lifelong reduction in the plasma level of LDL cholesterol is associated with a substantial reduction in the incidence of coronary events [37]. These findings have led to the development, testing and marketing of the powerful lipid-lowering PCSK9 inhibitors.

Whereas loss-of-function mutations in the *LDLR* are known to cause elevated levels of LDL cholesterol and premature cardiovascular disease, gain-of-function mutations in *LDLR* with a large effect on LDL cholesterol levels had not been described. Icelandic investigators analysed whole-genome sequencing data from 43,202 Icelanders [38]. Single-nucleotide polymorphisms and structural variants including deletions, insertions and duplications were genotyped using whole-genome sequencing-based data. LDL cholesterol associations were carried out in a sample of >100,000 Icelanders with genetic information. A 2.5-kb deletion (del2.5) overlapping the 3’ untranslated region of *LDLR* was discovered in seven heterozygous carriers from a single family [38]. Mean level of LDL cholesterol was 74% lower in del2.5 carriers than in 101,851 noncarriers, a difference of 2.48 mmol/L (96 mg/dL; *p* = 8.4 × 10^−8^). Del2.5 resulted in production of an alternative mRNA isoform with a truncated 3’ untranslated region [38]. Truncation leads to loss of target sites for microRNAs known to repress translation of *LDLR*. Lifelong very low levels of LDL cholesterol due to del2.5 did not appear to be detrimental to the health of carriers. Del2.5 is the first reported gain-of-function mutation in *LDLR* causing a large reduction in LDL cholesterol [38].

Gene regulatory networks (GRNs) control gene expression during development and adult life in animals and plants. Transcription factors and signalling molecules function as part of GRNs in which they control the expression of regulatory and all other genes by means of regulatory interactions. GRNs are organised as networks because each gene is regulated by many transcription factors and each transcription factor regulates many genes. Transcription factors represent the nodes of these networks and are essential for regulating gene expression [39]. Given the known sex differences in ischaemic heart disease, with women developing more stable atherosclerosis than men, key driver genes of sex-specific GRNs of atherosclerotic arterial walls of 160 women and age-matched men were investigated in the Stockholm–Tartu Atherosclerosis Reverse Network Engineering Task study [40]. Clear sex differences in GRN activity within the atherosclerotic tissues were found [40]. Genes more active in women were associated with mesenchymal and endothelial cells, whereas genes more active in men were associated with the immune system. Key drivers of GRNs active in females were predominantly found in plaque smooth muscle cells and affected by the Kruppel-like factor 4 transcription factor, involved in the regulation of proliferation, differentiation, apoptosis and somatic cell reprogramming. These results provide insights into the molecular mechanisms that underlie sex differences in atherosclerosis [40].

## 8. Challenges and Future Perspectives

Considerable challenges remain. Among these are the standardisation of genomic and deep-phenotyping methods and platforms, the definition of protocols for quality control, and the accounting for multiple testing inherent to large data analyses (Table 1) [11]. Machine learning is likely to assume an increasing role in developing predictive models, based on the complex interplay between genetic, deep phenotype and clinical variables, to stratify different levels of risk and responses to treatment. As a growing number of clinical trial data are now open to the public, the elaboration of different types of big data with machine technologies is becoming a reality; secondary analyses of combined clinical trial datasets may provide a powerful way of solving clinical questions with a new perspective [9]. Future objectives include: the development of statistical algorithms applied to dense phenotypic data to achieve comprehensive mapping of different phenogroups; the identification of useful biomarkers to guide drug dosing or other treatment strategies, and the identification of patient groups that differ in drug responsiveness; the definition of deep molecular crosstalk and biological networks (e.g., thrombosome, inflammasome, lipidome, fibrosome) underpinning different phenotypes at baseline and in response to injury and repair (Table 1) [12].

Moving from scientific discovery to practical implementation requires time, but transition from the concept of precision medicine as a disruptive innovation [12] to one of constructive coupling of refined diagnostics and treatment—already seen for cancer—is now occurring for ACS. Drastic potentiation is now necessary, with increased emphasis on stratified approaches in analysing multi-omic datasets.

## Figures and Tables

**Table 1:** Tools and challenges of ACS precision medicine.

Non-Omic Characteristics	Genomic and Extra-Genomic Mapping Tools	Future Challenges
● Demographics ● Presentation ● History ● Environment ● Diet ● Psychosocial ● Other risk factors ● Outcomes ● Medications ● Medical adherence ● Adverse events ● Imaging ● Traditional laboratory ● Histology ● Cytometry ● Immunophenotype	● Genome ● Epigenome ● Transcriptome ● Next-generation sequencing ● Metabolome ● Lipidome ● Glycome ● Proteome ● Inflammasome ● Thrombosome ● Fibrosome ● Immunophenome ● Metagenome/microbiome ● Interactome ● Radiomics	● Standardisation and quality control ● Accounting for big data multiple testing ● Identification of: - drivers of divergent response to treatment - biomarker-guided treatment strategies or dosing - biological networks/molecular crosstalk in health and disease ● Deep precision mapping combined with holistic medical approach

ACS: acute coronary syndromes.

**Table 2:** Established and investigational precision treatments of ACS.

	Criterion	Precision Treatment
**Established** **Treatments**	ST-segment elevation on ECG	Immediate coronary revascularisation of chest pain syndrome patients [4,20]
	Cardiac troponin elevation in blood	Early coronary angiography of non-ST-elevation ACS patients [4,20]
	GRACE mortality risk score >140	Intensified monitoring of ACS patients at enhanced risk of complications [4,20]
	PRECISE-DAPT bleeding risk score ≥25	Shortened DAPT duration in high bleeding risk ACS patients [4,20]
**Investigational** **Treatments**	Non-obstructive eroded coronary plaque at OCT	No PCI in ACS patients [21,22]
	No loss of function CYP2C19 gene variant	Clopidogrel instead of other P2Y12 inhibitors post ACS [23,24,25,26]
	RS1967309 variant of adenylate cyclase type 9 gene	Dalcetrapib (CETP inhibitor) for prevention of adverse events post ACS (ongoing DalGenE trial) [27]

ACS: acute coronary syndrome; CETP: cholesteryl ester transfer protein; CYP2C19: cytochrome P450 2C19; DAPT: dual antiplatelet therapy; GRACE: age, cardiac arrest at admission, blood pressure, heart rate, ECG changes, myonecrosis marker, creatinine, rales; PCI: percutaneous coronary intervention; OCT: optical coherence tomography; PRECISE-DAPT: age, creatinine clearance, haemoglobin, leukocyte count, prior bleeding.
